# Determining transgender identity: a discussion from legal, medical, and social perspectives

**DOI:** 10.3389/fpubh.2025.1643488

**Published:** 2025-09-03

**Authors:** Peng Sun, Youdan Hu, Fang Xie, Wei Li, Zhaoyi Guo

**Affiliations:** ^1^Department of Legal, Shenzhen Nanshan Maternity and Child Healthcare Hospital, Shenzhen, China; ^2^School of Public Health, Sun Yat-sen University, Guangzhou, China; ^3^Department of Laboratory Medicine, Maternity and Child Healthcare Hospital of Nanshan District, Guangzhou, China

**Keywords:** transgender, gender identity, legal recognition, medical ethics, social acceptance

## Abstract

As societal awareness of gender diversity continues to evolve, the legal and social recognition of transgender identities has emerged as a critical global issue. Transgender individuals encounter a range of legal challenges that necessitate collaborative efforts across various sectors for effective resolution. This paper examines the legal, medical, and socio-cultural practices related to transgender identity determination in China and other countries. It identifies key factors influencing the recognition of transgender identities and provides concrete recommendations for enhancing the rights of transgender individuals. By strengthening legal advocacy and education, refining legal frameworks, bolstering enforcement mechanisms, and fostering social inclusivity and diversity, we can create a more equitable, just, and supportive environment for transgender individuals.

## 1 Introduction

In the context of a rapidly evolving society, the conceptualization of gender increasingly reflects a state of “fluidity” rather than the traditional notion of “fixity.” Both academia and the broader public are recognizing the pressing need to address the identity affirmation and legal rights of sexual minority groups, including transgender individuals, bisexuals, homosexuals, and cross-dressers (1). These groups encounter numerous challenges related to legal identity recognition and social acceptance.

Globally, estimates suggest that the prevalence of transgender individuals ranges from approximately 1 to 3% of the total population, varying by country. In the United States, this prevalence is reported to be 1.6%, with a notable upward trend among younger generations. In populous nations such as India and China, the estimated number of transgender individuals is considerable. Reports indicate that in China, while around 400,000 individuals have sought gender-affirming surgeries, only about 1,000 have successfully undergone the procedure (2). The significant impact of traditional cultural norms, coupled with inadequate legal protections for transgender rights, has resulted in considerable challenges in their work, personal lives, and family dynamics.

This paper aims to analyze and discuss strategies for clarifying the issue of transgender identity affirmation through the enhancement of legal frameworks and the optimization of healthcare policies, ensuring the effective protection of their fundamental human rights.

## 2 Literature search and methodology

A comprehensive and systematic literature search was conducted from January 2013 to May 2025 to identify relevant academic, legal, and policy sources pertaining to transgender identity determination. The search strategy encompassed four major databases: PubMed for biomedical literature, Web of Science for interdisciplinary scholarship, CNKI (China National Knowledge Infrastructure) for Chinese-language sources, and HeinOnline for legal documents. A carefully designed Boolean search string was employed: (“transgender” OR “gender identity” OR “gender-affirming” OR “transsexual”) AND (“legal recognition” OR “law” OR “policy” OR “medical ethics” OR “social acceptance” OR “China”). To ensure comprehensive coverage, searches were conducted in both English and Chinese, and reference lists of included articles were manually screened to identify additional relevant sources.

The study established rigorous inclusion criteria consisting of: (1) peer-reviewed journal articles, authoritative legal reviews, official government reports, and methodologically sound NGO publications; (2) publications in either English or Chinese; (3) studies specifically addressing legal, medical, or social dimensions of transgender identity determination; and (4) works published between 2013 and 2025 to capture contemporary developments. Exclusion criteria systematically eliminated: (1) non-scholarly media articles, editorials lacking empirical evidence, and purely opinion-based commentaries; (2) studies unrelated to transgender identity recognition; and (3) duplicate publications.

Quality assessment was conducted using a dual-method approach: a modified Critical Appraisal Skills Programme (CASP) checklist was applied to qualitative and legal studies, while the GRADE system was used for quantitative and policy-impact analyses. Two independent reviewers evaluated each source for methodological rigor, transparency of legal reasoning, and definitional clarity, with any disagreements resolved through consensus discussion. All included studies were classified as high, moderate, or low quality based on this rigorous assessment process.

For data analysis, NVivo 14 software was employed to manage and code the collected materials. An initial coding framework was developed around four core thematic domains: legal frameworks, medical protocols, social acceptance, and ethical considerations. This framework underwent iterative refinement as new themes emerged during analysis. Inter-coder reliability was formally measured and maintained at Cohen’s *κ* ≥ 0.80, ensuring consistency in coding interpretations.

The final source selection process prioritized a balanced representation of jurisdictions, disciplines, and perspectives. International legal materials were included to facilitate cross-country comparisons, while domestic Chinese sources (including legal documents, policy reports, and local qualitative studies) provided essential cultural and legal context. Throughout the selection process, empirical studies were given precedence over purely descriptive accounts to strengthen the evidentiary foundation for subsequent analysis and policy recommendations.

## 3 Challenges faced in gender transition

### 3.1 Current situation of transgender individuals in China

In China, the process for transgender individuals to change their gender on identification documents is outlined in the Ministry of Public Security’s directive concerning gender change post-surgery (Public Security [2008] No. 478). This requires individuals to provide a gender assessment certificate from a certified tertiary hospital, along with notarized documentation or proof from a judicial authentication agency. Approval must be obtained from the relevant municipal public security department, after which local police stations can process the gender change. However, this procedure poses significant challenges. Obtaining the necessary gender assessment certificate requires individuals to present multiple documents, including proof of psychological evaluation, a written request for surgery, declarations from immediate family members, and a certificate of no criminal record. Only after all these documents are validated can the individual proceed with the surgery and related administrative tasks.

In one case from a Chinese city, a transgender patient sued a hospital for interrupting their gender-affirming surgery. The patient asserted that the hospital unilaterally halted the procedure, citing a lack of necessary documentation, which constituted a breach of contract. In another instance, a different hospital required the patient to provide proof of family consent and medical documentation from other institutions indicating a diagnosis of “gender dysphoria” before agreeing to perform the surgery. A significant concern among healthcare providers is whether gender determination should be based on chromosomal sex or the secondary sexual characteristics post-transition. Furthermore, there is a lack of established guidelines within the medical community regarding gender assessment, raising questions about the ability of hospitals to issue certificates while ensuring patient confidentiality. Due to the absence of clear legal frameworks, some transgender individuals who have undergone male-to-female surgery in Thailand have returned to China only to find that local hospitals can issue certificates confirming the surgery but do not provide a definitive gender designation. Consequently, these documents are deemed unacceptable by public security authorities. Moreover, hospitals have expressed both an unwillingness and an inability to furnish additional supporting documentation beyond the initial assessment.

The critical issue here is the “gender assessment certificate” issued by tertiary hospitals. Many institutions have responded by stating that they lack the qualifications for such assessments and are uncertain about how to issue a post-operative gender assessment certificate. This creates a cyclical problem: while legal gender change requires a gender assessment certificate, hospitals are neither equipped nor willing to provide a conclusive response, resulting in a deadlock (3).

As social beings, human gender expression reflects both biological and sociocultural dimensions. Although transgender individuals may achieve gender transition through surgical means, their chromosomal sex remains unchanged, limiting reproductive possibilities with the opposite sex. Their external appearance and functions may align with their affirmed gender. Importantly, transgender individuals often accept their target gender prior to surgery and seek to live socially in that role, aligning their social identity with their affirmed gender (4, 5). Thus, the core issue does not lie in the technicalities of gender determination but rather in societal acceptance and the absence of unified legal norms and procedures that are essential for protecting the rights of transgender individuals.

### 3.2 International perspectives

In the United States, policies regarding the recognition of transgender identity vary significantly across states. Some states impose stringent requirements for changing gender on identification documents, necessitating specific surgical proof. However, a majority of states permit transgender individuals to alter their gender on identification documents without undergoing surgery, typically requiring evidence of having undergone or being scheduled for gender-affirming treatment (6).

In Europe, many countries have reformed their legal frameworks governing the recognition of transgender identities in recent years. The United Kingdom, in particular, has taken significant steps to fully recognize the rights of transgender individuals to change their gender, even eliminating the requirement for surgical intervention.

In Asia, policies concerning the recognition of transgender identities vary widely. Thailand is relatively open regarding visibility for transgender individuals and the availability of gender-affirming surgeries, yet the legal process for gender change remains complex and requires medical documentation. In contrast, Japan mandates that individuals seeking to change their gender registration undergo a series of medical procedures, including sterilization, a policy that has faced widespread international criticism (7).

Overall, there is a growing trend internationally toward increased acceptance and tolerance of transgender individuals, gradually lowering barriers to legal gender change and significantly improving their legal status and quality of life (8).

### 3.3 Legal challenges

The rapid advancement of medical technology often outpaces legal frameworks, which tend to be more static due to their authoritative nature. As a result, legal protections for transgender rights remain in a state of flux, with varying degrees of clarity across different jurisdictions, frequently hindering the safeguarding of their rights.

Although the Ministry of Public Security issued guidelines addressing these issues as early as 2002 and 2008, clarifying the procedures for household registration changes for transgender citizens, the lack of effective enforcement (low policy efficacy) means that these guidelines do not adequately resolve practical issues. For example, the Chengdu Public Security Bureau treats “gender-affirming surgery certificates” as having equivalent validity to gender assessment certificates, a practice that clearly deviates from the Ministry’s regulations. The Ministry mandates that citizens undergoing gender-affirming surgery must provide a gender assessment certificate from a tertiary hospital, duly notarized, to effectuate a change in gender on household registration. However, health administrative departments have not developed corresponding strategies to address these issues, such as defining medical institutions qualified to conduct gender assessments or establishing standards for gender assessment for transgender individuals (8, 9). While the issue of gender change post-transition may appear straightforward on the surface, the Ministry’s responses apply only to specific cases and fall under administrative normative documents that do not possess the authority of higher-level regulations. Consequently, their legal efficacy and general applicability are questionable, as the recognition of citizens’ rights should be based on more authoritative normative documents.

### 3.4 Medical procedures and ethical issues

#### 3.4.1 Medical standards for gender-affirming surgery

In China, medical institutions performing gender-affirming surgeries must possess specific qualifications to ensure the safety and efficacy of these procedures. Such surgeries must adhere to strict protocols and standards established by the National Health Commission and relevant medical regulatory authorities. Key components of these standards include comprehensive psychological evaluations conducted prior to surgery, aimed at assessing the patient’s mental readiness for gender transition and their expectations regarding surgical outcomes. Additionally, hormone therapy is typically an integral part of the preoperative process, designed to help align the patient’s physical characteristics with their target gender. Long-term postoperative follow-up is essential for monitoring both physical health and psychological adjustment.

Despite the availability of certified medical facilities within China, many transgender individuals opt to undergo surgery abroad in countries such as Thailand, Australia, and the Netherlands, where advanced techniques and extensive experience are often found. However, significant disparities exist in surgical standards and postoperative care across different countries and institutions. A major challenge faced by Chinese transgender individuals returning home after surgery abroad is how to achieve effective gender recognition and identity confirmation in the absence of acceptable documentation from foreign medical facilities. Domestic laws and medical systems frequently require transgender individuals to provide a gender assessment certificate issued by a local tertiary hospital. Due to differences in international medical records and surgical standards, these foreign documents are often not recognized by domestic authorities, necessitating additional medical assessments to validate the legitimacy and medical effectiveness of their gender transition.

#### 3.4.2 Medical ethics

A critical ethical concern arises regarding the protection of individuals’ identities and privacy following gender-affirming surgery. Transgender individuals may encounter significant risks, such as identity theft, during the process of changing their gender and legal identity. Therefore, medical assessments and identity verification procedures must be meticulously controlled to ensure the legality and security of all documentation. This not only pertains to the protection of individual privacy but also relates to the broader maintenance of social order.

#### 3.4.3 Role and responsibilities of medical institutions

In the context of gender determination, the absence of comprehensive procedural standards often leads to medical personnel deferring responsibility, fearing unintended complications. This bureaucratic inertia results in inefficient administrative processes, leaving the legitimate needs of transgender individuals unmet. Many healthcare providers argue that the chromosomal sex of transgender individuals remains unchanged post-transition, which leads to reluctance in issuing necessary certificates.

However, if chromosomal sex is upheld as the sole criterion, it effectively negates the possibility of successful gender-affirming surgeries. Transgender individuals undergo these procedures to align their physical and psychological sexes, often enduring significant challenges in the process. From a humanitarian perspective, it is imperative to recognize the gender of individuals following surgery; failing to do so risks placing them in a precarious position of being perceived as neither male nor female. This can lead to substantial difficulties, including discrimination and an inability to safeguard their privacy rights (10, 11). Transgender individuals seek to alter their physical sex through gender-affirming surgery to align with their gender identity. These surgeries typically encompass a range of procedures, such as breast reconstruction and genital reconstruction.

For male-to-female transitions, the surgical alterations include the removal of the penis and testicles, realignment of the urethra to fit the new anatomical structure, and the construction of a neovagina utilizing flap surgery. The labia majora and clitoris are also reconstructed to simulate female genitalia. Conversely, for female-to-male transitions, procedures involve the removal of the uterus, ovaries, and vagina, along with the reconstruction of the penis, scrotum, and testicles. After these surgeries, the external genitalia of transgender individuals differ markedly from their assigned sex at birth, suggesting a potential approach for gender assessment agencies to issue gender assessment certificates based on the evaluation of external genitalia.

However, employing birth-assigned external genitalia for gender assessment raises significant ethical considerations. Every individual has the right to determine their own identity, including gender identity. Conducting gender assessments, such as chromosomal evaluations, at birth could undermine an individual’s future exploration of their gender identity. Additionally, birth-assigned gender assessments involve sensitive personal information, necessitating stringent confidentiality measures to prevent psychological and social harm in the event of information breaches. Currently, medical institutions also exhibit shortcomings in protecting the privacy of transgender individuals and providing psychological support. There is an urgent need to enhance legal frameworks to safeguard the rights and well-being of transgender individuals.

### 3.5 Legal considerations for establishing transgender identity

International human rights law, grounded in the Universal Declaration of Human Rights and subsequent treaties, mandates that states protect the legal rights of lesbian, gay, bisexual, transgender, and intersex (LGBTI) individuals. Every person, regardless of gender, sexual orientation, or gender identity, is entitled to protections under international human rights law, including the right to life, personal security, privacy, freedom from torture, protection against arbitrary arrest and detention, freedom from discrimination, and rights to free speech, marriage, and peaceful assembly. Despite a gradual increase in social tolerance and acceptance of the LGBTI community in China, transgender individuals still face low visibility and acceptance in society. Many struggle to engage fully in social life and report suicidal tendencies. A survey of 1,309 transgender individuals in China indicated a lifetime prevalence of suicidal ideation at 56.4% (732 out of 1,298 respondents) and a suicide attempt rate of 16.1% (209 out of 1,298 respondents) (12). These issues often arise not from the individuals themselves, but from a lack of familial and societal support, pervasive discrimination, and the immense pressure caused by complex and opaque legal processes for gender recognition (13).

To address these challenges, it is imperative to establish clear legal norms delineating specific procedures and standards for gender recognition, along with pilot implementations in designated areas. This approach would help mitigate ambiguity and arbitrariness in legal application. Key considerations could include: (1) individuals must be at least 20 years old and have experienced a desire for gender transition for no less than two years, affirming their acceptance of their target gender; (2) they must undergo evaluation by a psychiatrist to confirm the absence of mental illness; (3) individuals who are married should consult with their spouse regarding the dissolution of their marriage prior to transitioning; and (4) there must be no contraindications to surgical procedures.

Moreover, safeguarding the legal rights of transgender individuals requires explicit legal prohibitions against discrimination based on gender identity across all sectors, including employment, education, healthcare, and social services (14, 15). Establishing legal aid and consultation services is also essential to assist transgender individuals in navigating legal issues and asserting their rights. It is recommended that a coordinating body composed of representatives from public security and health departments be formed to facilitate the gender recognition and rights protection of transgender individuals. The implementation of this mechanism is crucial for ensuring coherence and effectiveness in policy development and execution, as well as for addressing current deficiencies in interdepartmental collaboration (16, 17).

## 4 Discussion and recommendations

The determination of transgender identity represents a complex and multifaceted issue that requires careful consideration from legal, medical, ethical, and sociocultural perspectives. Our comprehensive analysis of China’s current framework, situated within a broader global context, reveals systemic challenges that demand urgent and comprehensive reform. The existing legal recognition process in China, as stipulated by the Ministry of Public Security’s directive (Public Security [2008] No. 478), establishes a rigid protocol that requires transgender individuals to not only undergo gender-affirming surgery but also obtain a gender assessment certificate from designated tertiary hospitals. While this system was ostensibly created to provide a clear pathway for legal gender recognition, in practice it has created numerous institutional barriers and procedural inconsistencies that place disproportionate burdens on transgender applicants.

Qualitative research and firsthand accounts from Chinese transgender individuals (12, 18) consistently highlight several critical pain points within the current system. First, the bureaucratic processing times are excessively protracted, often stretching into years rather than months, creating prolonged periods of legal limbo for applicants. Second, there is significant inconsistency in how guidelines are interpreted and applied across different administrative units, with some local authorities demanding additional documentation not required by central policies. Third, and perhaps most damaging psychologically, is the requirement for repeated disclosures of deeply personal medical histories to various officials throughout the application process. These lived experiences find striking parallels in ethnographic studies conducted in Thailand and policy analyses from European nations (19, 20), demonstrating that even in jurisdictions with relatively progressive legal frameworks on paper, the gap between policy formulation and practical implementation can substantially undermine the intended benefits of legal recognition.

The medical dimension of gender recognition presents equally complex challenges that warrant careful examination. While international trends clearly show a gradual shift toward depathologizing transgender identity - as evidenced by Argentina’s groundbreaking self-declaration model and the UK’s reforms to its Gender Recognition Act - China’s continued reliance on surgical interventions as an absolute prerequisite for legal recognition creates significant accessibility issues. Medical institutions frequently decline to issue the required gender assessment certificates, citing either lack of clear operational guidelines or concerns about potential legal liability (21). This institutional hesitancy has created a paradoxical situation where the very documents needed to facilitate legal recognition remain effectively inaccessible to the majority of applicants. Furthermore, the requirement for gender-affirming surgery presents substantial financial barriers, as these procedures are typically not covered by public health insurance and can cost upwards of 100,000 RMB for comprehensive treatment. This economic burden disproportionately affects lower-income transgender individuals, effectively creating a two-tier system where legal recognition becomes contingent upon one’s financial means.

Our comparative analysis of global models yields several important insights that could inform potential reforms in the Chinese context. Argentina’s self-declaration system, implemented alongside robust anti-discrimination protections, has demonstrated measurable improvements in mental health outcomes among transgender populations, including statistically significant reductions in stigma experiences and lower rates of suicidal ideation (22). The Argentine model is particularly noteworthy because it completely decouples legal gender recognition from medical interventions, instead relying on a simple administrative process where individuals can update their identity documents through a notarized affidavit. At the opposite end of the spectrum, Japan’s recently overturned sterilization requirement exemplifies how medically invasive prerequisites can perpetuate human rights violations despite superficial procedural clarity (23, 24). The Japanese case is instructive in demonstrating how even developed nations can maintain regressive policies until challenged through judicial review. The UK’s intermediate approach, which combines elements of medical evaluation with strong equality protections under the Equality Act 2010, offers another potential model for consideration, though its relatively low uptake (with fewer than 5,000 Gender Recognition Certificates issued since the law’s implementation in 2004) suggests the need for complementary public education initiatives to improve accessibility (25–27).

Building on this comprehensive evidence base, we propose the following integrated recommendations for reforming China’s gender recognition framework:

First and foremost, legal reforms should focus on establishing nationally standardized procedures that significantly reduce current bureaucratic burdens while maintaining necessary administrative integrity. This could involve adopting a modified self-declaration model that draws lessons from Argentina’s experience but is carefully adapted to China’s specific administrative and cultural context. Such a system might incorporate a mandatory reflection period and basic information requirements to prevent potential abuse while still dramatically simplifying the overall process. The reformed procedures should be codified in properly promulgated administrative regulations rather than remaining as internal directives, giving them greater legal force and consistency in application across different regions.

Second, medical policy harmonization must address the current ambiguities in assessment protocols. A phased approach could be considered, initially maintaining some medical involvement but shifting the focus from surgical requirements to comprehensive psychological evaluations conducted by qualified professionals. This transition would align with emerging best practices in transgender healthcare (11) while addressing concerns about maintaining appropriate professional oversight. The National Health Commission should work with professional medical associations to develop clear, standardized guidelines for these evaluations, including provisions for recognizing medical documentation from reputable international providers when applicants have undergone treatment abroad.

Third, privacy protections and anti-discrimination safeguards require substantial strengthening to prevent the secondary victimization of transgender individuals during documentation processes. Specific measures should include strict limitations on who can access an individual’s gender history, prohibitions against unnecessary disclosure requirements in employment and education settings, and clear penalties for violations. These protections should be complemented by public education campaigns to increase understanding and acceptance of transgender issues among the general population and key professional groups such as healthcare providers, educators, and law enforcement personnel.

Implementation of these reforms should proceed through carefully designed and monitored pilot programs in selected provinces, allowing for evidence-based refinement before nationwide rollout (28, 29). These pilots should incorporate robust mechanisms for ongoing evaluation of multiple outcome measures, including mental health indicators, employment discrimination patterns, and social integration metrics, drawing on methodologies employed in similar international studies (28). The pilot phase should last sufficiently long (at least 2–3 years) to allow for proper assessment of both short-term and medium-term effects.

Crucially, the entire policy development process must actively incorporate the lived experiences of transgender communities through structured consultation mechanisms. This could take the form of regular stakeholder forums, community advisory panels, and targeted surveys to ensure that reforms address real-world needs rather than abstract bureaucratic imperatives. Particular attention should be paid to including voices from less visible segments of the transgender community, including rural residents, older individuals, and those from lower socioeconomic backgrounds.

This comprehensive approach recognizes that meaningful and sustainable reform requires simultaneous action across multiple domains: streamlining administrative procedures, modernizing medical protocols, enhancing legal protections, and fostering cultural change through targeted education initiatives. By grounding these recommendations in both rigorous empirical evidence and nuanced qualitative insights from affected communities, we aim to contribute to the development of a more equitable and effective gender recognition framework that aligns with international human rights standards while remaining responsive to China’s specific sociocultural context and developmental needs.

The proposed reforms would represent a significant step forward in protecting the rights and dignity of transgender individuals in China, while also positioning the country as a regional leader in progressive gender policies. Properly implemented, such changes could substantially improve quality of life for transgender citizens, reduce public health burdens associated with mental health disparities, and contribute to a more inclusive and harmonious society overall. Future research should focus on monitoring the implementation and outcomes of any reforms, with particular attention to how they affect different subgroups within the transgender population and interact with other areas of law and policy.
